# Bad Habits that Stick: An Investigation into Adhesive Medical Tape Use Practices and Beliefs

**DOI:** 10.1017/ash.2024.136

**Published:** 2024-09-16

**Authors:** Julia Fischer, Sarah Haessler

**Affiliations:** Baystate Medical Center; Baystate Health

## Abstract

**Background:** Medical tape is one of the most ubiquitous resources in the hospital. Although tape is advertised by manufacturers as a single patient-use item, half-used rolls are a common sight in hospitals. Tape is often manipulated by un-sanitized and ungloved hands and comes in close contact with patient skin. Medical tape has the potential to be a source of hospital-acquired infection as it has been documented to be colonized with pathogens ranging from MRSA to Rhizopus. Despite infection risk, currently the only clinical guidelines of tape use are outlined in the Centers for Medicare & Medicaid Services guidance for Hemodialysis patients issued in 2008 that requires “tape should be dedicated to a single patient and discarded after use” as hemodialysis patients are at higher risk of infection. However, there is a lack of standards in the practice of tape use across hospital systems. **Methods:** To understand the current practices and beliefs of tape use at our institution, we developed a standardized survey to query individuals from various roles (RN, Physician, Patient Care Technicians, respiratory therapists, phlebotomists) across all patient care areas at a 746-bed academic, tertiary care center. **Results:** 52 units were surveyed, including 225 employees. Qualitative analysis revealed a wide variety of uses for medical tape for patient care, with venipuncture, securing IVs, and wound dressings being the most common. Only 1.4% of individuals reported single use of tape rolls. 54% of individuals reported tape use behaviors that carry an elevated risk for inoculation of pathogens. 70% of individuals reported that tape was discarded after the patient was discharged from their respective area. These practices did not change across procedure-heavy areas such as the Emergency Department or the Operating Rooms, in fact only 22% of individuals surveyed reported single use of tape in these areas. Beliefs about tape use varied: 95% of individuals agreed that a roll of tape could be used multiple times on a single patient, and 52% of individuals agreed that a roll of tape could be used on multiple patients. **Conclusions:** Tape use practices varied across hospital units, indicating the need for standardized policies for tape use and storage. Beliefs about tape not being a single-use item were consistent across the hospital and suggest that education and culture change efforts are needed to decrease the risk for hospital-acquired infections from improper medical tape use.

